# Editorial Notes

**Published:** 1927

**Authors:** 


### Editorial Notes.

**Death of Sir Isambard Owen.**

THE death of Sir Isambard Owen, formerly Vice-Chancellor of the University of Bristol, deprives the academic world of a notable authority. It was no common achievement to have spent a considerable part of a long life as a physician in London and then made such a mark in University administration. The University of Bristol owes more than it as yet realises to Owen's administrative ability and guidance during its infancy. Medicine is, perhaps, no bad training ground for acquiring a knowledge of the handling of human affairs.

**Resignation of Miss E. E. P. MacManus, R.R.C.**

WE regret to have to record the resignation of Miss MacManus from the Matronship of the Royal Infirmary, though we offer her our warmest congratulations on her appointment to be Matron of Guy's Hospital.

Miss MacManus came to Bristol in 1923 from Guy's Hospital, where she had held many appointments, including that of Assistant Matron. Her career has been a distinguished one, and her wide experience has rendered her an outstanding personality in the nursing world. Before the war she had nursed in Egypt, both privately and at the Kasr-el-Aini Hospital. Her army work in France had brought her to such close quarters with the Germans, that the Casualty Clearing Station, to which she was attached at Noyon, was at one time believed to have actually been captured during the retreat of the 5th Army in 1918.

Miss MacManus takes with her to Guy's the good wishes of everyone in Bristol who knew her. She maintained an exceptionally high standard of efficiency in nursing at the Royal Infirmary, and won the affection of her nurses in a remarkable degree by her sympathy and consideration.

**Complimentary Dinner to Dr. Lily Baker.**

ON February 4th a complimentary dinner was organised by some of her women friends at the Royal Infirmary to Dr. Lily Baker, on the occasion of her appointment to the honorary staff of the Infirmary. The dinner was held at Fortt's and was attended by a hundred guests. Dr. Baker's health was proposed by Lady Barrett, Professor Winifred Cullis and Dr. Letitia Fairfield. Their speeches showed that the art of after-dinner oratory is not neglected by women.

**Fiftieth Anniversary of the Foundation of University College.**

The Fiftieth Anniversary of the Foundation of University College, Bristol, was celebrated on December 4th, 1926, by the Association of Alumni. We hope in our next issue to publish an article by Mr. Richardson Cross dealing with the history of the College, and more especially with the various movements which led up to its foundation. Mr. Cross's personal recollections of the early days of University College will have exceptional interest, as he was connected with the College from its start and later formed one of the Committee that obtained the Charter for the University of Bristol.

